# Multidisciplinary diagnosis of interstitial lung disease in Zambia using videoconferencing

**DOI:** 10.11604/pamj.2022.41.183.33450

**Published:** 2022-03-08

**Authors:** Thijs Hoffman, Wouter van Es, Jan Grutters, Kondwelani Mateyo

**Affiliations:** 1 Department of Pulmonology, St. Antonius Hospital, Nieuwegein, The Netherlands,; 2 Department of Radiology, St. Antonius Hospital, Nieuwegein, The Netherlands,; 3 Department of Pulmonology, Division of Heart and Lungs, University Medical Centre, Utrecht, The Netherlands,; 4 Department of Internal Medicine, University Teaching Hospital, Lusaka, Zambia

## To the editors of the Pan African Medical Journal

The diagnosis and management of interstitial lung diseases (ILD) are usually performed in the context of a multidisciplinary team [1]. This is associated with improved diagnostic confidence and is considered to be the gold standard for ILD diagnosis [2]. A recent study found the practice of multidisciplinary team diagnosis was widespread across the world [3]. However, this study had few responses from Africa, and it was noted that the frequency of formal multidisciplinary team meetings was lower in African centres compared to other centres.

Overall, there are few epidemiological data available on the prevalence of ILD in Africa [4, 5]. Factors that potentially influence prevalence of ILD in African countries are higher rates of occupational exposure to silica [6], lower prevalence of cigarette smoking [7], and a higher prevalence of tuberculosis and human immunodeficiency virus infection [8, 9]. Here we present the results of a collaboration between University Teaching Hospital in Lusaka, Zambia, and the ILD Centre of Excellence at St. Antonius Hospital in Nieuwegein, The Netherlands.

Starting in December 2018 we have held bimonthly videoconferences, lasting approximately one hour. One or more pulmonologists and one or more radiologists (with >20 years of experience in ILD) from St. Antonius Hospital are present. The patients are presented by the registrars, medical students, or a pulmonologist from a University Teaching Hospital. The chest CT-scan, which was sent to St. Antonius Hospital in advance, is then described by the radiologist. The pulmonologists then discuss relevant findings and differential diagnoses. Advice is formulated with regard to further investigations and/or patient management. After the conference, a written report of the cases that were discussed is sent to the University Teaching Hospital. For this study, approval was obtained from the Zambian medical ethics committee (UNZABREC reference number 1960-2021).

In total, 41 unique patients have been discussed in the course of ten videoconferences (Table 1 and Table 2). After multidisciplinary discussion and (in some cases) additional diagnostic investigations, twenty-one patients most likely had an interstitial lung disease. This included seven patients with pulmonary fibrosis (one with likely idiopathic pulmonary fibrosis, four with connective-tissue disease associated to pulmonary fibrosis, one with combined pulmonary fibrosis and emphysema, and one with drug-induced pulmonary fibrosis), as well as six patients with sarcoidosis (histopathological confirmation in three patients), two patients with hypersensitivity pneumonitis, two patients with silicosis, two patients with lymphangioleiomyomatosis, one patient with organizing pneumonia, and one patient with childhood-ILD.

**Table 1:** overview of patients with a presumed ILD diagnosis that have been discussed at the videoconferences

Sex/age, relevant medical history, exposures	Previous treatment for TB	Chest CT findings	Histopathology	Most likely diagnosis (differential)
Pulmonary fibrosis				
F/44	Yes	fNSIP, dilated oesophagus	NA	CTD-ILD (SSc, Sjogrens, idiopathic fNSIP)
M/69, HIV	No	fNSIP, possibly fDIP, dilated PT	NA	CTD-ILD (fDIP, CHP, idiopathic fNSIP)
M/58	No	fNSIP, enlarged PT	NA	CTD-ILD (idiopathic fNSIP)
F/59, presumed sarcoidosis in 2017	No	Fibrosis in lower lobes consistent with fNSIP; no lymphadenopathy	NA	CTD-ILD (idiopathic fNSIP)
F/72, HF	No	Pulmonary fibrosis, indeterminate for UIP	NA	IPF (CHP, CTD-ILD, idiopathic fNSIP)
M/72, 40 PY	No	Emphysema in upper fields with some fibrotic changes in lower fields	NA	CPFE
F/31, MTX for RA	No	fNSIP	NA	MTX-related lung disease (CTD-ILD)
Sarcoidosis				
M/59, 5PY, mineral dust	Yes	Diffuse subpleural and perifissural nodular abnormalities	Non-caseating granulomas (TBB)	Sarcoidosis (pneumoconiosis)
F/65, CVA	No	Hilar and mediastinal lymphadenopathy, perifissural intrapulmonary nodules	NA	Sarcoidosis (TB)
F/42	Yes	Mediastinal lymphadenopathy, multiple consolidations, cavitating lesion in right upper lobe	Non-specific chronic inflammatory infiltrates (TBB)	Sarcoidosis, complicated by pulmonary aspergilloma
F/19,	Yes	Mediastinal and hilar lymphadenopathy, intraparenchymal nodules in lung parenchyma also perifissural; hepatosplenomegaly	Mostly non-caseating granulomas (LN)	Sarcoidosis
M/22	No	Bilateral hilar and mediastinal lymphadenopathy with subpleural and perihilar pulmonary nodules	Non-caseating granulomas (TBB)	Sarcoidosis
M/47, pneumonia 3 months ago	No	Diffuse pulmonary nodules in the perihilar subpleural regions; bilateral hilar and mediastinal lymphadenopathy	Chronic inflammation with lymphocytic infiltration (TBB)	Sarcoidosis
Other				
F/66, CKD, AF	No	Some fibrosis in the apices, diffuse mosaic attenuation with air-trapping; elevated right hemidiaphragm	NA	CHP
M/51, parrot, grain silos	No	Diffuse ground glass and mosaic attenuation	NA	HP (DIP, NSIP, DAH)
F/38, recurrent pneumothoraces	No	Cystic lung disease, suggestive of LAM	NA	LAM
F/43, HIV	No	Diffuse cystic lung disease, perivascular and subpleural	NA	LAM (BHD, LIP)
M/47, electrician in a mine, joint pains and positive ANA	Yes	Diffuse nodular abnormalities also perifissural, no lymphadenopathy	NA	Caplan syndrome
M/29, gemstone cutter	No	Diffuse nodular abnormalities predominantly in the upper fields, some consolidative changes, subpleural thickening, enlarged PT	NA	Silico-proteinosis
M/53, exposure to fumes as mechanic	Yes	Multiple ground glass areas without fibrosis	NA	OP, possibly due to toxic exposure at work (infection)
M/15	Yes	Diffuse cystic lung disease with fibrosis and traction bronchiectasis, most consistent with surfactant gene mutation	NA	Childhood ILD, possibly related to surfactant gene mutation

F = female, M = male, fNSIP = fibrotic non-specific interstitial pneumonia, HIV = human immunodeficiency virus, TB = tuberculosis, CVA = cerebrovascular accident, PY = packyears, MTX = methotrexate, RA = rheumatoid arthritis, HF = heart failure, CKD = chronic kidney disease, AF = atrial fibrillation, NA = not available, LN = lymph node, fDIP = fibrosing desquamative interstitial pneumonia, PT = pulmonary trunk, (C)HP = (chronic) hypersensitivity pneumonitis, DAH = diffuse alveolar haemorrhage, SSc = systemic sclerosis; CTD-ILD = connective-tissue disease associated interstitial lung disease, CPFE = combined pulmonary fibrosis and emphysema, LAM = lymphangioleiomyomatosis, TBB = transbronchial biopsy, BHD = Birt-Hogg-Dubé syndrome, LIP = lymphoid interstitial pneumonia, PE = pulmonary embolism, ANA = antinuclear antibodies

**Table 2:** overview of patients with a presumed non-ILD diagnosis that have been discussed at the videoconferences

Sex/age, relevant medical history, exposures	Previous treatment for TB	Chest CT findings	Histopathology	Most likely diagnosis (differential)
Related to infections or aspiration				
F/34	Yes	Bilateral pectoral lymphadenopathy, left axillary lymphadenopathy, no intrapulmonary abnormalities	Caseating granulomas (LN)	Infection (TB, possibly sarcoid-like reaction after infection)
F/47, HIV	No	Airway disease	NA	Infection
F/68	No	Ground glass with minimal fibrosis: fNSIP	NA	Infection with some post-infectious sequelae (CTD-ILD, drug-induced ILD)
F/29, DVT	No	Multiple large nodules, consolidation in right lower lobe	NA	Infection (OP, vasculitis)
M/61, 50 PY, amiodarone	No	Mediastinal and hilar lymphadenopathy, diffuse ground glass and consolidations	NA	Infection (amiodarone toxicity, acute HP)
F/64	No	Bronchiectasis in right lower lobe, post-infectious sequelae in left lower lobe, dilated oesophagus	NA	Chronic aspiration
M/65	No	Bilateral consolidations, cavitating lesions in the left lung, dilated oesophagus	NA	Chronic aspiration (vasculitis, fungal infection)
M/28, 4 PY, construction worker	Yes	Dilated trachea with diverticula (>30mm), dilated main bronchi, diffuse cystic bronchiectasis	NA	Mounier-Kühn syndrome
Cardiac or pulmonary vascular disease				
M/50	No	Normal	NA	Cardiac disease (gastro-esophageal reflux)
F/63, cardiomyopathy	No	fNSIP or hemosiderosis, dilated PT	NA	Hemosiderosis due to chronic heart failure (fNSIP)
F/65	Yes	Bilateral consolidations and pleural effusions	NA	Heart failure complicated by infection
F/51	No	Ground glass in mosaic pattern, honeycombing, consolidations, dilated PT	NA	CTEPH (CTD-ILD, CHP, or FPF)
M/68, HIV, right ventricular mass being analyzed	No	Some emphysema in upper lobes, dilated PT, thrombus at wall of right pulmonary artery	NA	PE or pulmonary arterial sarcoma
Airway diseases				
M/62, HIV, 42 PY	Yes	Paraseptal emphysema with some fibrosis, cavitary lesion with a mass in the left upper lobe, tracheal thickening on the right	NA	Emphysema with aspergilloma and postinfectious changes in trachea
M/48	No	No abnormalities	NA	Asthma
F/29	No	Diffuse mosaic attenuation, airway disease	NA	Asthma
M/46, HIV	No	Spiculated mass in left upper lobe, several small nodules in right upper lobe	NA	Asthma, possibly lung cancer or post-infectious changes in left upper lobe
Other				
M/31, pneumonia 6 months ago	No	Somewhat dilated pulmonary trunk, otherwise no abnormalities	NA	Unknown (methemoglobulinemia, sickle cell disease)
M/52	No	No abnormalities	No abnormalities (TBB)	Unknown (sarcoidosis)
F/61	Yes	Intrapulmonary calcifications, hepatomegaly, hyperintense liver	NA	Unknown (Amyloidosis, hemochromatosis, pneumoconiosis, diffuse pulmonary ossification)

F = female, M = male, HIV = human immunodeficiency virus, TB = tuberculosis, CVA = cerebrovascular accident, PY = packyears, OP = organizing pneumonia, DVT = deep vein thrombosis, MTX = methotrexate, RA = rheumatoid arthritis, HF = heart failure, CKD = chronic kidney disease, AF = atrial fibrillation, NA = not available, LN = lymph node, fDIP = fibrosing desquamative interstitial pneumonia, PT = pulmonary trunk, (C)HP = (chronic) hypersensitivity pneumonitis, DAH = diffuse alveolar haemorrhage, CTD-ILD = connective-tissue disease associated interstitial lung disease, TBB = transbronchial biopsy, FPF familial pulmonary fibrosis

In patients who did not have an interstitial lung disease, the most likely diagnoses included infection or post-infectious sequelae in five patients, lung damage due to chronic aspiration in two patients, pulmonary vascular disease in two patients, heart failure in three patients, asthma in three patients, emphysema in one patient and Mounier-Kühn syndrome in one patient [10]. In three patients, no clear diagnosis could be made. Of note, eleven out of 41 patients had previously been treated for presumed tuberculosis (in the absence of positive sputum cultures). This included three patients who were eventually diagnosed with sarcoidosis (all patients had negative tuberculosis diagnostics on bronchial washings, and sarcoidosis was confirmed by histopathology in two out of three patients).

Several issues were encountered that make diagnosis and treatment of ILD more difficult in Zambia. First, the quality of radiological imaging is not always optimal, which makes interpretation more difficult. However, in the last two years, we have seen a clear improvement in image quality. Second, some diagnostic tests are not (easily) available, including diffusion capacity of the lung for carbon monoxide, open lung biopsy, and nail fold capillary microscopy. Third, the patient delay is often longer, which makes treatment less effective in several types of fibrotic lung disease. Fourth, not all treatment options are (easily) available in Zambia, including antifibrotic agents, second- and third-line immunosuppressive agents, and lung transplantation. Fifth, patient follow up is often more difficult, as some patients have to travel much longer to come to the hospital. Finally, this study illustrates the dangers of giving pragmatic anti-tubercular treatment based on chest X-rays and symptoms, as many patients were treated for presumed tuberculosis, but turned out to have an ILD. This can hopefully be ameliorated by an increased availability of CT-scanners and increased awareness of ILD.

In the future, registries of ILD diagnoses in sub-Saharan Africa can be used for comparing prevalence of different types of ILD and the response to treatment. Registration of ILD diagnoses would provide insight into local prevalence of specific diseases, such as work-related ILD (e.g., pneumoconiosis), and this might aid with the implementation of preventive measures.

**Conclusion:** in conclusion, the whole spectrum of ILD can be seen in sub-Saharan Africa. International collaborations could contribute to improving diagnosis and care for patients with ILD and other rare and/or complex diseases all over the world, even though they only require relatively small investments of time and infrastructure from both parties.
